# Risk factors of failure results after double-bundle reconstruction with autogenous hamstring grafts for isolated posterior cruciate ligament rupture cases

**DOI:** 10.1038/s41598-024-56953-y

**Published:** 2024-03-14

**Authors:** Yudai Morita, Takuya Tajima, Nami Yamaguchi, Takuji Yokoe, Makoto Nagasawa, Tomomi Ota, Kouki Ouchi, Etsuo Chosa

**Affiliations:** Division of Orthopedic Surgery, Department of Medicine of Sensory and Motor Organs, Faculty of Medicine, University of Miyazaki, 5200 Kihara, Kiyotake, Miyazaki 889-1692 Japan

**Keywords:** Posterior cruciate ligament, Hamstring, Posterior tibial translation, Gravity sag view, Medical research, Risk factors

## Abstract

Posterior tibial translation (PTT) after double-bundle posterior cruciate ligament (PCL) reconstruction has sometimes occurred. Purpose of this study is to identify the risk factors for postoperative PTT after double-bundle PCL reconstruction with a hamstring autograft. Comparing the results of bilateral gravity sag view (GSV) at 12 months after surgery, over 5-mm PTT was defined as ‘failure’ in this study. Of 26 isolated PCL reconstruction cases, over 5-mm PTT was seen in 7 cases (group F: 9.57 ± 1.28 mm), and 19 cases had less than 5 mm (group G: 2.84 ± 1.29 mm). Age, sex, body mass index (BMI), preoperative GSV, posterior slope angle of the tibia, anterolateral bundle (ALB) and posteromedial bundle (PMB) graft diameters, and tibial tunnel diameter were evaluated. The two groups were compared with the 2 × 2 chi-squared test, the Mann Whitney U-test, and Spearman’s rank correlation coefficient. Multivariate logistic regression analysis was also performed to determine the risk factor. Statistical significance was indicated as p < 0.01 for correlation with postoperative PTT, and as p < 0.05 for all other comparisons. Mean age (group G 31.8 ± 12.5 vs group F 34.9 ± 15.9 years), sex (male/female: 15/4 vs 3/4), BMI (25.6 ± 4.6 vs 24.9 ± 3.9 kg/m^2^), preoperative GSV (11.3 ± 2.2 vs 11.6 ± 2.9 mm), PMB diameter (5.37 ± 0.33 vs 5.36 ± 0.48 mm), and tibial tunnel diameter (9.32 ± 0.58 vs 9.29 ± 0.49 mm) showed no significant differences. ALB diameter was significantly greater in group G (7.0 ± 0.5 mm) than in group F (6.5 ± 0.29 mm; p = 0.022). There was also a significant difference in posterior tibial slope angle (group G 9.19 ± 1.94 vs group F 6.54 ± 1.45, p = 0.004). On Spearman rank correlation coefficient analysis, ALB diameter GSV (correlation coefficient: − 0.561, p = 0.003) and posterior tibial slope angle (correlation coefficient: − 0.533, p = 0.005) showed a significant correlation with postoperative PTT. Multivariate logistic regression analysis showed that ALB diameter (OR 19.028; 95% CI 1.082–334.6; p = 0.044) and posterior slope angle of tibia (OR 3.081; 95% CI 1.109–8.556; p = 0.031) were independently associated with postoperative PTT, respectively. In double-bundle PCL reconstruction with hamstring, smaller ALB graft diameter and lower (flatted) tibial slope angle were considered risk factors for postoperative PTT.

## Introduction

The posterior cruciate ligament (PCL) is the largest and strongest intraarticular ligament of the knee joint, as well as the main posterior stabilizer^1,2^. The PCL is composed of two bundles, the larger anterolateral bundle (ALB) and the smaller posteromedial bundle (PMB)^3,4^. Isolated PCL injuries are rare, with a reported annual incidence of 2 per 100,000 persons^3,5^. Most cases of PCL injuries present concurrently with other knee ligament injuries, such as injuries to the anterior cruciate ligament (ACL), medical collateral ligament, and posterolateral structures^2,6^.

Treatment options for isolated PCL injuries are still controversial and include conservative treatment and surgical treatment. Outcomes of PCL reconstruction have been reported to be not as good as those following ACL reconstruction^7–9^. There also have been described radiographic progression of osteoarthritis and decreased functional outcomes as the time from injury increased for isolated PCL tears treated nonoperatively^10–12^. Recently, surgical treatment such as reconstruction for isolated PCL injuries has been improving, and better outcomes compared with nonoperative management have also been reported^13,14^. Several reconstruction techniques have been described. Considering the factors of PCL reconstruction procedures, graft selection, bone tunnel placement, fixation method, fixation devices, knee angle and tensioning at the fixation, and postoperative rehabilitation protocols are important^5,15^. One of the most important factors in PCL reconstruction is the number of bundles^14,16^.

As reported by biomechanical studies, the ALB and PMB perform in a codominant manner, and these roles theoretically would not be restored by single-bundle PCL reconstruction, suggesting that an anatomical double-bundle PCL reconstruction may be able to provide more closely restore native kinematics than the single-bundle reconstruction procedure^17^.

Unfortunately, graft failure or posterior tibial translation (PTT) after double-bundle PCL reconstruction with soft tissue graft cases has sometimes occurred. Some studies reported that levels of side-to-side PTT after double-bundle PCL reconstruction measured by an arthrometer or radiographs were 2.4 mm to 3.9 mm, or 4 mm to 5 mm more than the native knee^2,18^. Residual posterior sagging was already occurred after 3 months postoperatively was also reported^19^. These changes after PCL reconstruction were reported that mechanical factors during postoperative rehabilitation, including the gravity of the patient’s shank weight, knee flexion, or hamstring contraction may facilitate larger graft elongation, tunnel enlargement, and consequently greater postoperative increases in the PTT in the early postoperative term^19–21^. Tachibana et al. also introduced that preoperative grade 3 injury was independently associated with residual posterior sugging^19^. However, the risk factors for postsurgical PTT after PCL reconstruction have not been fully elucidated. Previously, age, sex, and graft size or diameter were found to be significantly associated with graft failure after ACL reconstruction with hamstring grafts^22,23^.

The purpose of this study was to determine the risk factors for PTT after double-bundle PCL reconstruction with an autogenous hamstring graft to isolated PCL injuries. It was hypothesized that age, sex, and size of the hamstring autograft contribute to PTT after double-bundle PCL reconstruction.

## Results

Patient characteristics was shown in Table 1. Participants had a mean age of 32.7 ± 13.2 years. The sex ratio was 18 male and 8 female. The mean BMI was 25.4 ± 4.4 kg/m^2^. The mean leg symmetry index in knee extension and flection was 88.8 ± 17.6% and 93.4 ± 22.1%, respectively. The mean preoperative GSV was 11.4 ± 2.3 mm. The mean femoral ALB and PMB diameter was 6.87 ± 0.50 mm and 5.37 ± 0.36 mm, respectively. The mean tibial tunnel diameter was 9.31 ± 0.55 mm. The mean posterior slope angle on the tibia was 9.31 ± 0.55°. 11 cases were complicated with meniscus injury, 3 cases with periarticular fracture. The mean PTT value at 12 M after surgery was 4.6 ± 3.3 mm.

**Table 1:** Characteristics of participants (N = 26).

	Value
Age (years)	32.7 ± 13.2
Sex (male/female)	18/8
BMI (kg/m^2^)	25.4 ± 4.4
Leg symmetry index in knee extension (%)	88.8 ± 17.6
Leg symmetry index in knee flexion (%)	93.4 ± 22.1
Preoperative GSV (mm)	11.4 ± 2.3
Femoral ALB tunnel diameter (mm)	6.87 ± 0.50
Femoral PMB tunnel diameter (mm)	5.37 ± 0.36
Tibial tunnel diameter (mm)	9.31 ± 0.55
Posterior slope angle of the tibia (degree)	8.48 ± 2.16
Complication	
Meniscus injury (cases)	11
Periarticular fracture (cases)	3

*BMI* body mass index, *GSV* gravity sag view, *ALB* anterolateral bundle, *PMB* posteromedial bundle.

Of these 26 cases, posterior laxity greater than 5 mm was seen in 7 cases (group F: 9.57 ± 1.28 mm), and it was less than 5 mm in 19 cases (group G: 2.84 ± 1.29 mm). The mean age (group G; 31.8 ± 12.5 vs group F; 34.9 ± 15.9 years), BMI (25.6 ± 4.6 vs 24.9 ± 3.9 kg/m^2^), preoperative gravity sag view (GSV) (11.3 ± 2.2 vs 11.9 ± 2.9 mm), PMB diameter (5.37 ± 0.33 vs 5.36 ± 0.48 mm), and tibial tunnel diameter (9.32 ± 0.58 vs 9.29 ± 0.49 mm) showed no significant differences between group G and group F (Table 2). The mean leg symmetry index showed no significant difference in knee extension (91.5 ± 15.2% vs 81.4 ± 22.7%), while significantly difference was observed in knee flexion (97.3 ± 23.0% vs 82.8 ± 16.5%, p = 0.035). The ALB diameter (7.00 ± 0.50 mm) of group G were significantly larger than in group F (6.50 ± 0.29 mm, p = 0.022). The posterior slope angle of tibia was significantly higher in group G than that of group F (9.19 ± 1.94° vs 6.54 ± 1.45°, p = 0.004).

**Table 2:** Comparison of patient data between two groups.

	Group G	Group F	p value
	(N = 19)	(N = 7)	
Age (years)	31.8 ± 12.5	34.9 ± 15.9	0.866
Sex (male/female)	15/4	3/4	0.149
BMI (kg/m^2^)	25.6 ± 4.6	24.9 ± 3.9	0.735
Leg symmetry index in knee extension (%) at 12 M after surgery	91.5 ± 15.2	81.4 ± 22.7	0.231
Leg symmetry index in knee flexion (%) at 12 M after surgery	97.3 ± 23.0	82.8 ± 16.5	0.035*
Preoperative GSV (mm)	11.3 ± 2.2	11.6 ± 2.9	0.955
Femoral ALB tunnel diameter (mm)	7.00 ± 0.50	6.50 ± 0.29	0.022*
Femoral PMB tunnel diameter (mm)	5.37 ± 0.33	5.36 ± 0.48	0.821
Tibial tunnel diameter (mm)	9.32 ± 0.58	9.29 ± 0.49	0.866
Posterior slope angle of the tibia (degree)	9.19 ± 1.94	6.54 ± 1.45	0.004**
Complication			
Meniscus injury (cases)	7	4	
Periarticular fracture (cases)	3	0	

*BMI* body mass index, *GSV* gravity sag view, *ALB* anterolateral bundle, *PMB* posteromedial bundle, *M* months.

*< 0.05.

**< 0.01.

On Spearman rank correlation coefficient analysis, ALB diameter showed a significant correlation to the postoperative GSV (correlation coefficient: − 0.561, p = 0.003). The posterior slope angle of tibia was also significant correlation to postoperative GSV (correlation coefficient: − 0.533, p = 0.005) (Table 3).

**Table 3:** Correlations with postoperative GSV.

	Correlation coefficient	p value
Age	0.085	0.681
Sex (male/female)	0.347	0.077
BMI	− 0.023	0.91
Leg symmetry index in knee extension	− 0.21	0.302
Leg symmetry index in knee flexion	− 0.302	0.134
Preoperative GSV	0.112	0.587
ALB diameter	− 0.561	0.003**
PMB diameter	− 0.052	0.8
Tibial tunnel diameter	− 0.075	0.716
Posterior slope angle of tibia	− 0.533	0.005**

*BMI* body mass index, *GSV* gravity sag view, *ALB* anterolateral bundle, *PMB* posteromedial bundle.

*< 0.01.

**Figure 1:**
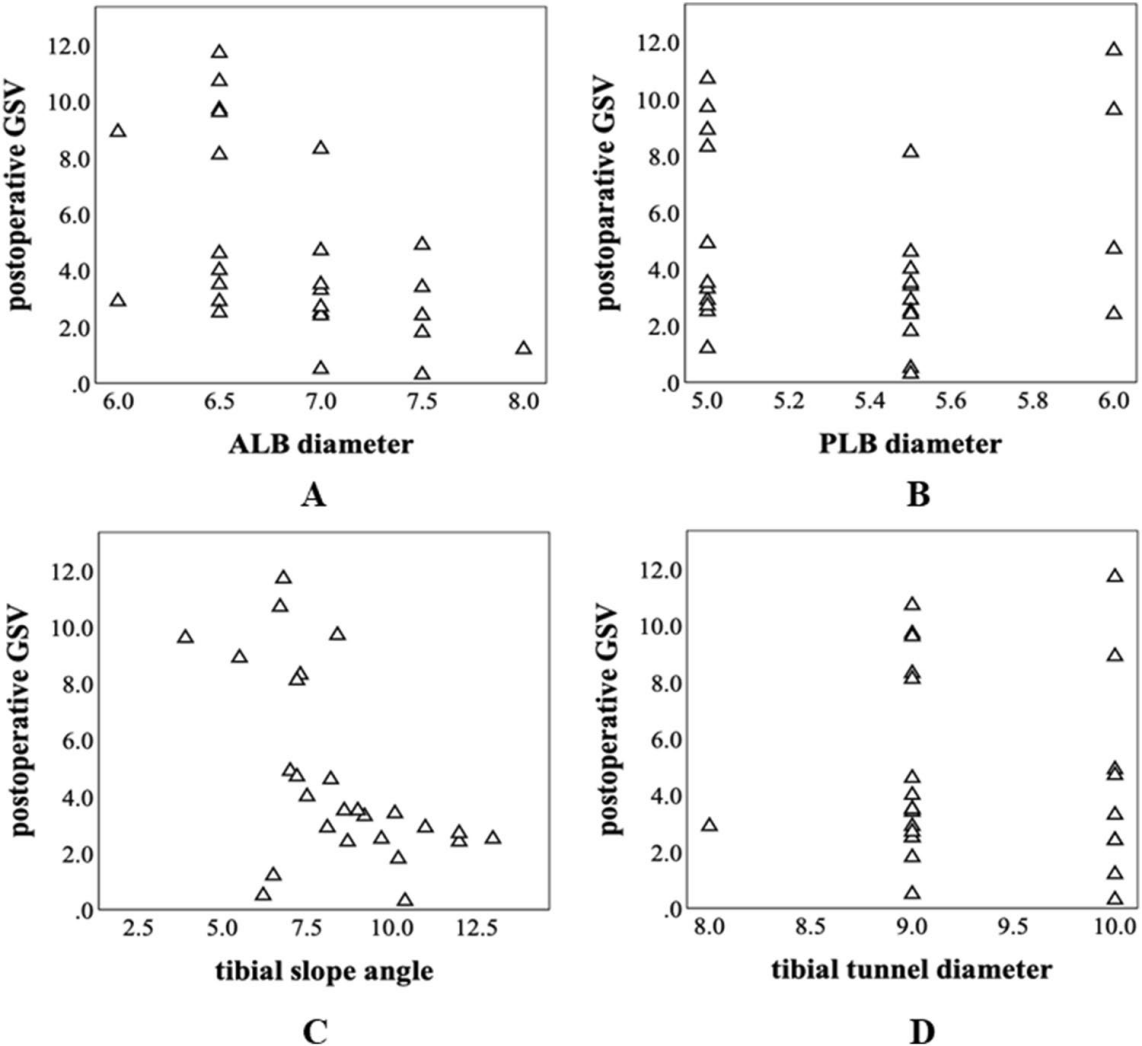
Proportions and correlations of postoperative GSV. (**A**) ALB diameter, (**B**) PMB diameter, (**C**) tibial slope angle, (**D**) tibial tunnel diameter.

The proportions and correlations of postoperative GSV and ALB, PMB diameter, tibial slope angle, and tibial tunnel diameter are shown in Fig. 1.

ALB tunnel diameter (odds ratio (OR) 19.028; 95% confidence interval (CI) 1.082–334.6; p = 0.044) and posterior slope angle of tibia (OR 3.081; 95% CI 1.109–8.556; p = 0.031) were independently associated with postoperative PTT from the multivariable logistic regression analysis (Table 4).

**Table 4:** Multivariate logistic regression analysis of risk factors for postoperative posterior laxity.

	Regression coefficient	SE	p value	OR	95% CI
Age	− 0.018	0.034	0.600	0.982	0.919–1.050
BMI	0.035	0.105	0.742	1.035	0.842–1.273
Leg symmetry index in knee extension	0.036	0.028	0.206	1.036	0.981–1.095
Leg symmetry index in knee flexion	0.039	0.985	0.159	1.040	0.985–1.098
Preoperative GSV	− 0.050	0.189	0.783	0.952	0.653–1.419
ALB diameter	2.945	1.463	0.044*	19.028	1.082–334.6
PMB diameter	0.090	1.249	0.943	1.093	0.095–12.661
Posterior slope angle of tibia	1.125	0.521	0.031*	3.081	1.109–8.556

*BMI* body mass index, *GSV* gravity sag view, *ALB* anterolateral bundle, *PMB* posteromedial bundle.

*< 0.05.

**Figure 2:**
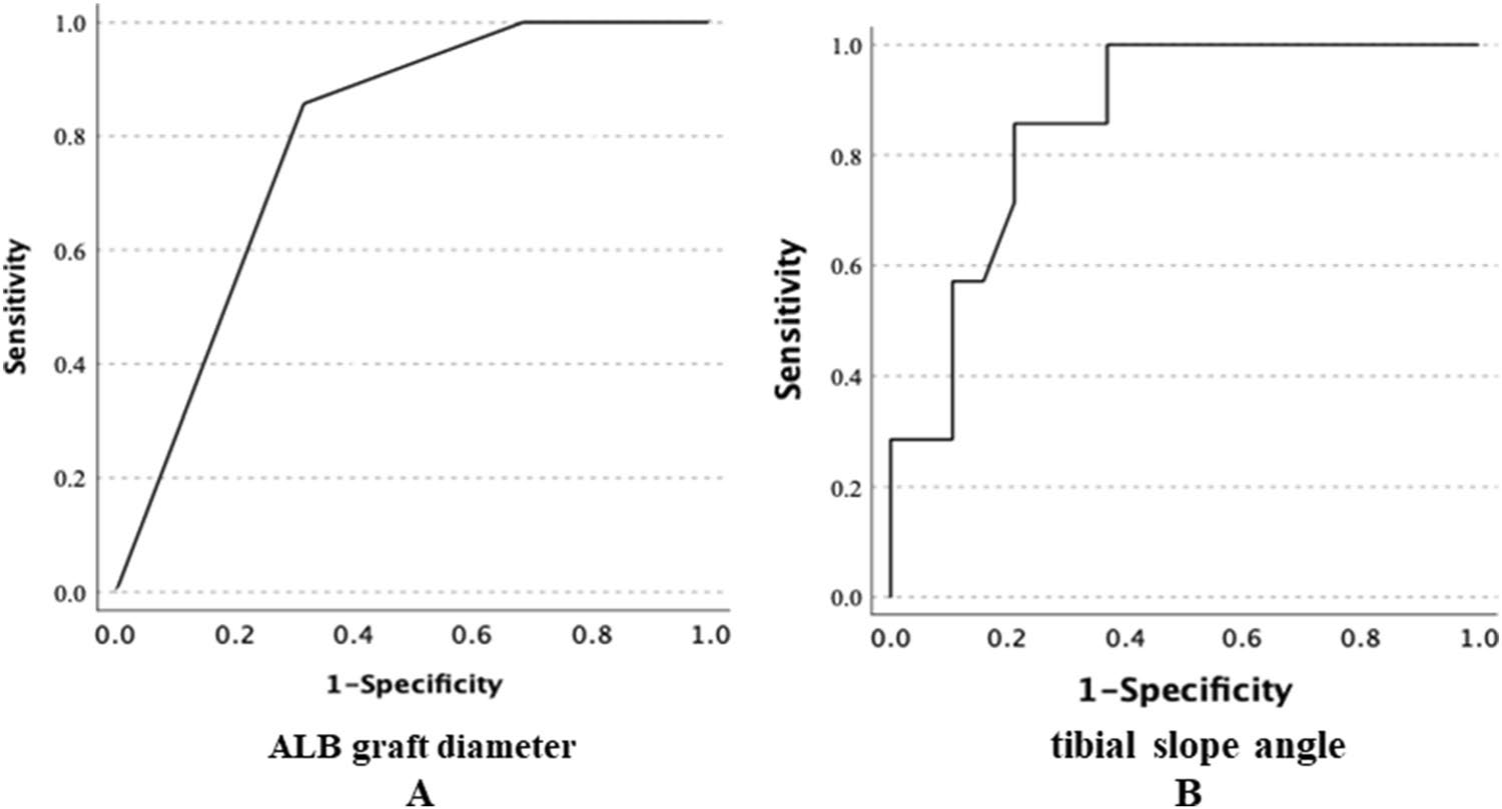
Receiver operating characteristics (ROC) curve of ALB graft diameter (**A**) and tibial slope angle (**B**). The area under the ROC curve was 0.793 (p = 0.001; 95% CI 0.617–0.969; SE 0.09), and the cutoff point value was 6.5 mm (sensitivity, 85.7%; specificity, 68.4%) for ALB diameter. And the area under the ROC curve was 0.861 (p < 0.001; 95% CI 0.718–1.003; SE 0.073), and the cutoff point value was 7.3 degree (sensitivity, 85.7%, specificity, 78.9%) for tibial slope angle.

Additionally, the receiver operating characteristic (ROC) analysis revealed a cutoff value of 6.5 mm of ALB diameter (sensitivity, 85.7%; specificity, 68.4%; 95% CI 0.617–0.969; p = 0.001) and 7.3 degree of posterior slope angle of tibia (sensitivity, 85.7%, specificity, 78.9%; 95% CI 0.718–1.003; p < 0.001) for the postoperative PTT as the threshold for differentiating between 2 groups those with and without postoperative PTT (Fig. 2).

## Discussion

The most important findings of the present study were that the diameter of the ALB graft and posterior slope angle of tibia were significantly associated with PTT after double-bundle PCL reconstruction with hamstring autografts. Namely, smaller ALB graft and flatted or shallowed tibial slope were considered as risk factors for postoperative PTT.

It is well known that the PCL functions as one of the main stabilizers of the knee joint and serves primarily to resist excessive posterior translation of the tibia relative to the femur^1,24,25^. Fox et al. have reported that the ALB provided the primary restraint to posterior tibial translation when the knee is flexed to 90°, and the PMB functions as the primary restraint to posterior tibial translation with the knee near full extension, as well as a secondary restraint to knee rotation^26^. Race et al. reported that majority of the strength of the PCL comes from the ALB, since the tensile strength of the ALB is 1620 N, whereas the tensile strength of the PMB is 258 N^27^. From these findings, the ALB is considered as the most important factor preventing posterior tibial re-translation after double-bundle PCL reconstruction at knee flexion of 90°.

Previously, several options have been described for graft selection: an Achilles tendon with bone plug allograft, quadriceps tendon allograft, bone-patellar tendon-bone (BPTB) graft for larger ALBs, and a semitendinosus tendon autograft and allograft, tibialis anterior allograft, tibialis posterior allograft for smaller PMBs^2,28^. Several surgical procedures and graft sizes were also introduced previously. Markolf et al. used an 11-mm BPTB graft for the ALB and an additional 8-mm BPTB graft for the PMB^29^. Pache et al. preferred an 11-mm Achilles allograft for the ALB graft, and a tibialis anterior allograft 7 mm in diameter for PMB^3^. Using allografts of sufficient size for ALB and PMB grafts might be better. However, in some countries, including Japan, allografts are not available for surgery. BPTB is one of the common grafts used for knee cruciate reconstruction. This type of graft has shown good stability, with bone-to-bone healing expected^30^. On the other hand, the disadvantages of using BPTB were also reported previously, including donor site morbidity, reduced knee extension strength, anterior knee pain, and so on^31,32^. In addition, Yoo et al. measured the geometry of the patellar tendon with knee magnetic resonance imaging, reporting that the mean patellar tendon width of male Korean adults (n = 142, height 175.7 ± 5 cm) had a proximal width of 30.3 mm and a distal width of 24.0 mm, and female adults (n = 30, height 162.6 ± 4.9 cm) had widths of 27.5 mm and 21.5 mm, respectively^33^. Oikawa et al. also reported in a cadaveric study that the mean proximal, central, and distal patellar tendon widths were 29.9 mm, 27.3 mm, and 25 mm, respectively^34^. Therefore, an over-8-mm BPTB autograft is larger than one-third of the patellar tendon width for Asian patients due to their physique. Milankov et al. reported patellar tendon rupture in 1.8% and patellar fracture after harvesting the BPTB in 0.45%^35^. They recommended that, to minimize the risk of patellar fracture, no more than a 25- to 30-mm length of the patella and no more than 9 to 10 mm of its width should be removed^35^. Moreover, Lin et al. concluded that a hamstring tendon autograft may be a better choice for transtibial tunnel PCL reconstruction compared with a patellar tendon autograft due to the lower incidence of anterior knee pain, squatting pain, kneeling pain, and osteoarthritic change^36^. It is well known that the advantage of using soft tissue grafts such as the hamstrings include easy control of the size and length for graft preparation. For these above reasons, we use hamstring autografts for double-bundle PCL reconstruction.

However, as already mentioned above, the ALB is considered the most important factor preventing posterior laxity after double-bundle PCL reconstruction at knee flexion of 90°. We usually harvested both a semitendinosus tendon graft and a gracilis tendon from the ipsilateral knee. Sometimes, a triple-folded or quad-folded semitendinosus tendon could not reach a sufficient diameter for an ALB graft in small physique people such as Asian patients. In these cases, a smaller diameter ALB graft may lead to postoperative PTT. When the harvested hamstring grafts were smaller (cutoff value was 6.5 mm), harvesting an additional graft source to make a larger size graft or converting the surgical procedure from double-bundle PCL reconstruction to other procedures should be considered.

Recently, several authors have reported the relationship between posterior tibial slope angle and PCL injury. Schatka and colleague reported that a high tibial slope was significantly correlated with increased posterior tibial translation^37^. On the other hand, Bernhardson et al. reported that PCL graft forces increased as tibial slope decreased (flattened) when loaded, and they concluded that the effect of tibial slope on PCL grafts was the same as that which has been noted clinically, and a flat tibial slope should be considered a factor when evaluating the cause of failed PCL reconstructions^38^. Gwinner et al. also reported that the flatted posterior tibial slope was associated with a significantly greater persistent PTT^39^. In the present study, decreased posterior slope angle of tibia was one of the optimal risk factors for postoperative PTT. Furthermore, the posterior tibial slope angle of the failure group was more decreased compared with the good group with significance. Although, we did not perform the biomechanics investigation in this study, our results support the previous reports of Bernhardson and Gwinner^38,39^.

Previously, preoperative grade 3 injury was the optimal risk factor associated with postoperative PTT has been reported^19^. It was possible that preoperative grade 3 injury induces other soft tissue laxity such as articular capsule. However, there was no significantly difference of preoperative GSV between failure group and good group in the present study. In addition, there remains controversy concerning the graft fixation angle. Both AL and PM bundles were fixed at 90°of knee flexion according to Kimura’s report in this series^40^. Tachibana et al. had been reported that both grafts were fixed at 0° of knee flexion^19^. On the other hand, Kennedy et al. recommended that PM graft should be fixed at 0° and the AL graft should be fixed at 90°at knee flexion^4^. These differences of graft fixation angle may influence postoperative PTT.

### Limitations

Several limitations must be taken into consideration with respect to the present study. First, the study had a small sample size and, second, it was not randomized. Third, the minimum follow-up period was 12 months after surgery. However, sequential change in PTT after surgery was confirmed in 3 months, and there was no significantly development between 3, 6, 12, and 24 moths has been reported^19^. Fourth, the details of the mechanisms and timing of posterior laxity after surgery were obscure. However, despite these limitations, the present study may contribute to providing important information for double-bundle PCL reconstruction with a hamstring autograft.

## Future perspectives

Based on this study, we recommend that if the harvested hamstring grafts were smaller (cutoff value was 6.5 mm), harvesting an additional graft source to make a larger size graft or converting the surgical procedure from double-bundle PCL reconstruction to larger single bundle PCL reconstruction should be considered. Also, patients with flat tibial slopes in chronic tears or revision PCL reconstruction cases should be evaluated closely for the possible need of a first-stage or concurrent slope-increasing tibial osteotomy. Yang et al. reported that using anterior opening wedge high tibial osteotomy to steepen the posterior tibial slopes and a larger tibial tunnel angles may be a promising surgical strategy^41^. In the future perspectives, prospective studies are required to evaluate the postoperative PTT by changing the technique to a larger single bundle PCL reconstruction for patients with a smaller AM bundle, and to investigate the efficiency and safety of the osteotomy for those with a flat tibial slope.

## Materials and methods

### Patients

The present study was conducted in January 2021, involving patients who were diagnosed with symptomatic PCL rupture and underwent double-bundle PCL reconstruction with autogenous hamstring grafts at our institute. The study design was reviewed and approved (Accession No. 0-0875, January 21.2021) by the Committee for Ethics of University of Miyazaki and informed consent was obtained from all subjects and/or their legal guardians. The procedures followed were in accordance with the ethical standards of the responsible committee on human experimentation (institutional and national) and with the Helsinki Declaration of 1975, as revised in 2013^42,43^. Information regarding the conduct of this study was disclosed, and research subjects were provided an opportunity to refuse inclusion in this study. The patients who did not want to take part were not enrolled in this study. The study involved a retrospective, observational design, with data collected from April 2006 to January 2021. Inclusion criteria were follow-up of at least 12 months after double-bundle PCL reconstruction with full clinical data available including the GSV X-ray examinations, and knee extension and flexion muscle strengths. Exclusion criteria consisted of revision surgery, any prior knee surgery, multiple ligament injuries such as anterior ligament involvement as indicated by the Lachman test^44^, abnormal varus/valgus laxity, and concomitant treatments for articular cartilage defects, such as osteochondral autologous transplantation. Cases with a follow-up period of less than 12 months and single-bundle reconstruction cases were also excluded. Three senior orthopaedic surgeons who have been experienced in knee ligamentous surgery performed full physical examinations of each ligament around the knee joint.

**Figure 3:**
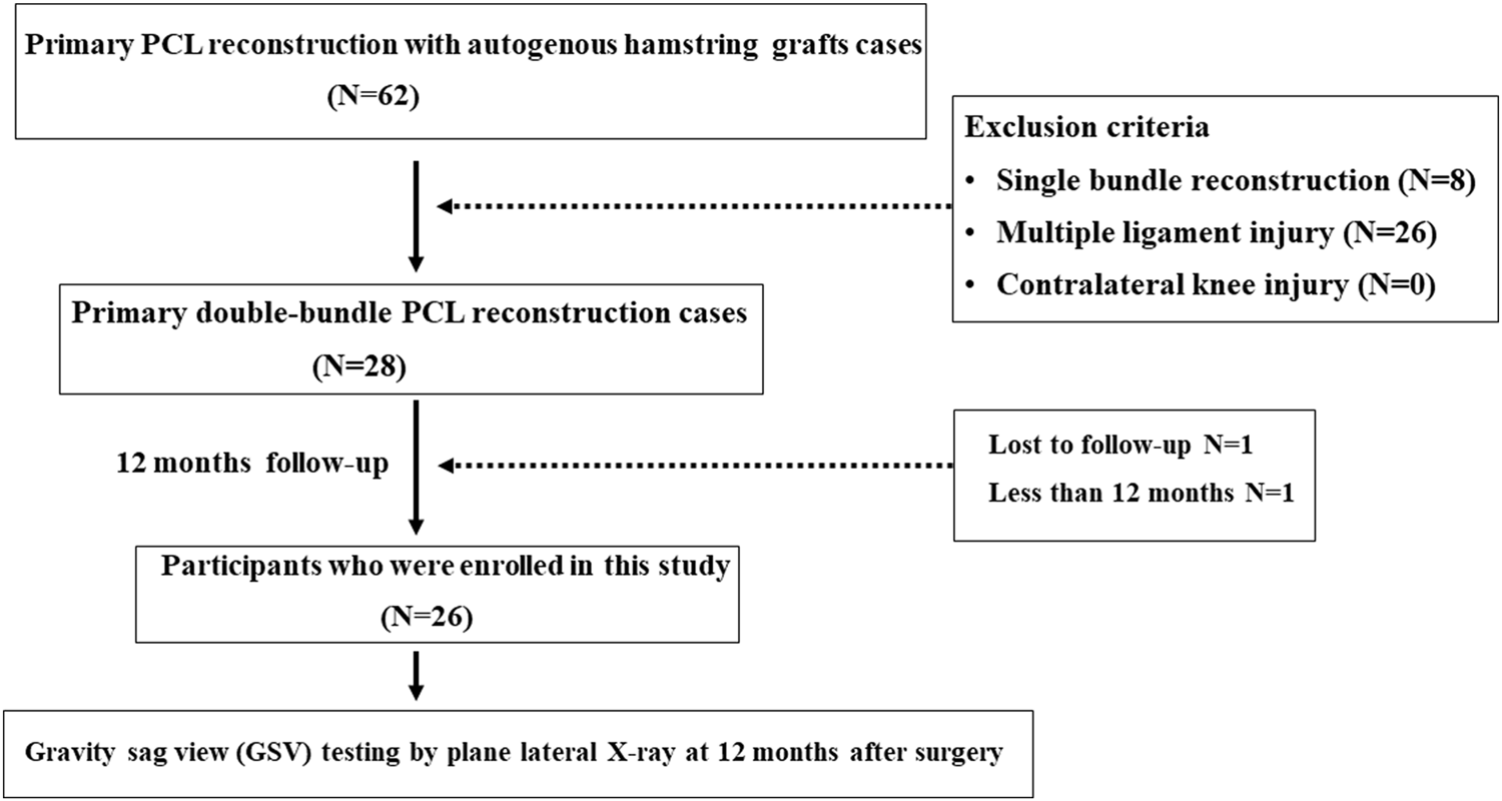
Participant flowchart. Of 62 cases, multiple ligament-involved cases, single-bundle PCL reconstruction cases, and cases lost to follow-up and with follow-up less than 12 months after surgery were excluded. Finally, 26 patients were enrolled in this study. Posterior tibial translation over 5 mm, which was measured by GSV, was seen in 7 cases, and it was less than 5 mm in 19 cases.

During the investigation period, 62 cases underwent PCL reconstruction. Of these 62 cases, 26 multiple ligament cases and 8 single-bundle PCL reconstruction cases were excluded. Single cases that were lost to follow-up and had follow-up of less than 12 months from PCL reconstruction were also excluded. Therefore, 26 patients met the inclusion criteria and were enrolled in this study (Fig. 3).

### Surgical procedure

**Figure 4:**
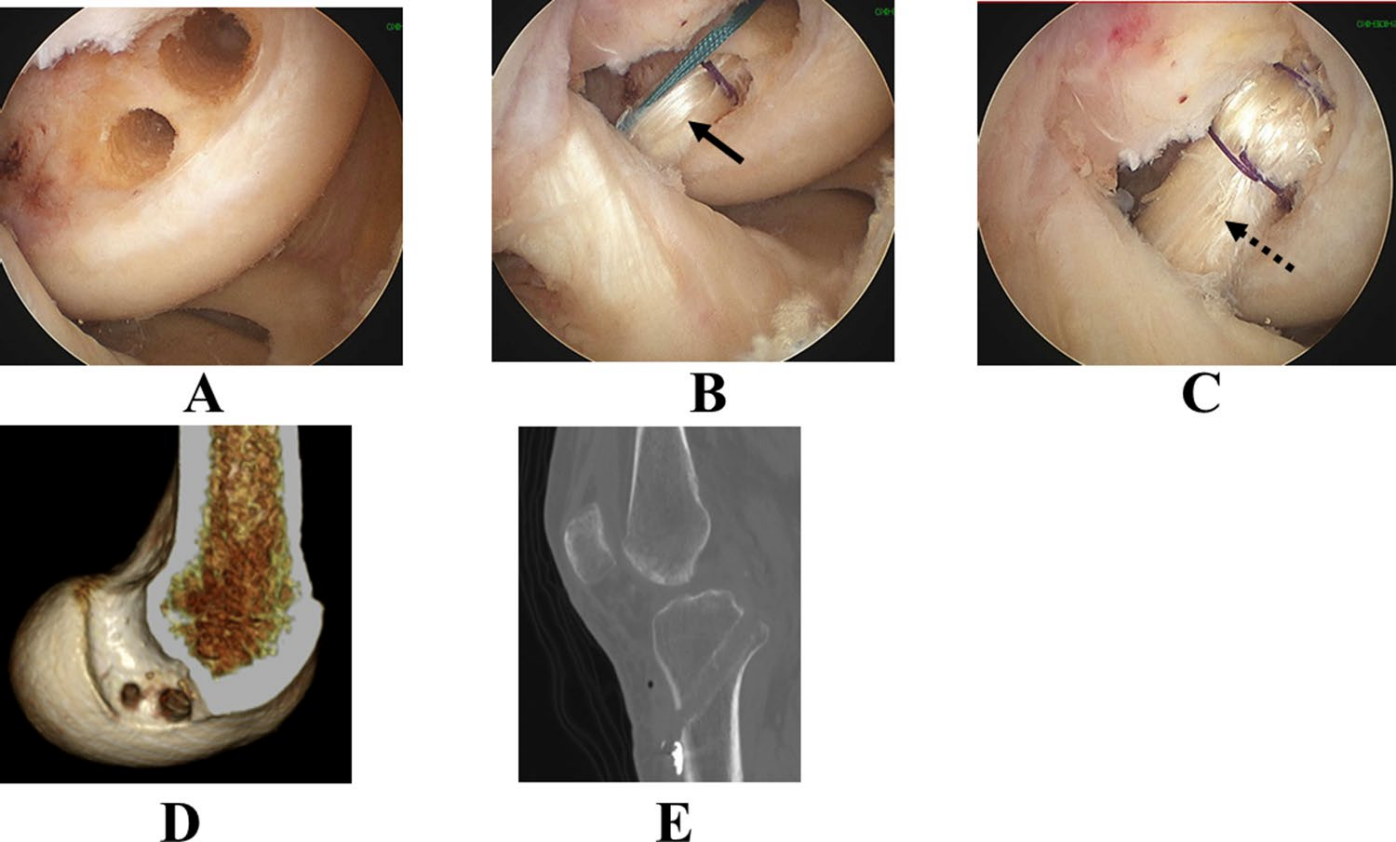
Surgical procedure of double-bundle PCL reconstruction. (**A**) Two bone tunnels are created at the femur. (**B**) The PMB bundle is inserted (arrow). (**C**) The ALB bundle is inserted (dotted arrow). (**D**) 3D-computed tomography findings of the femur. (**E**) Computed tomography findings of the tibia. One bone tunnel is created. (*PCL* posterior cruciate ligament, *PMB* posteromedial bundle, *ALB* anterolateral bundle).

**Figure 5:**
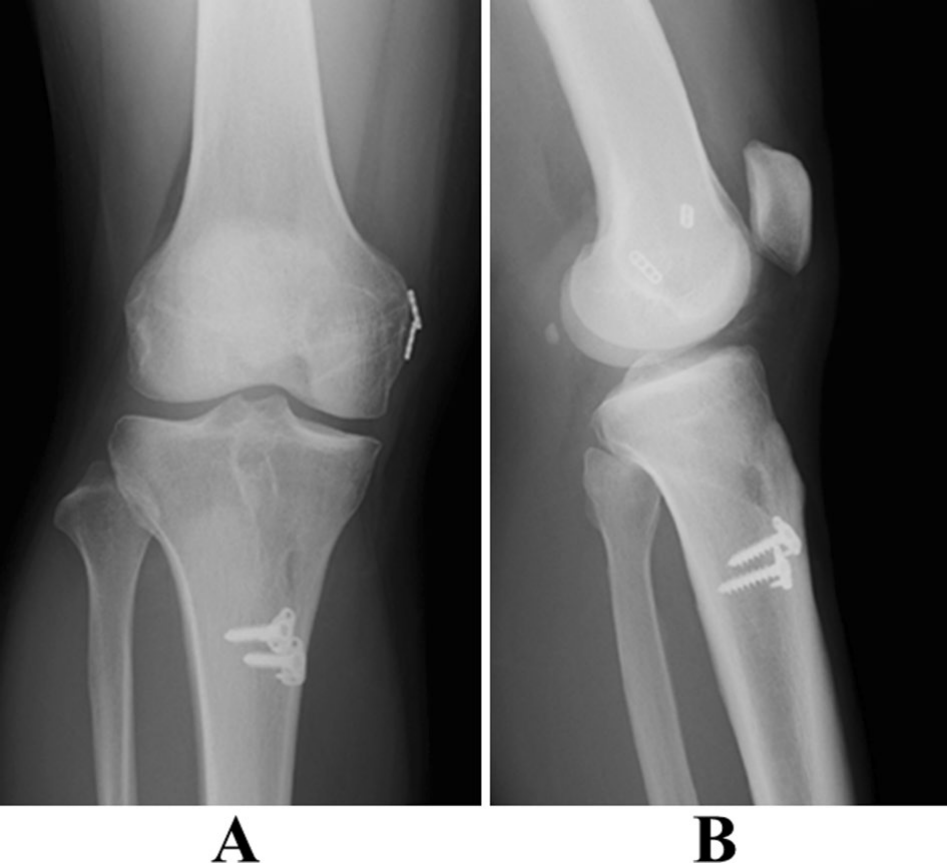
Postoperative X-ray findings. (**A**) Antero-posterior view. (**B**) Lateral view. The EndoButton-CL of the femoral side was flipped on the femoral cortical surface. The anterolateral and posteromedial graft were fixed to the tibia with a double-spike plate system.

A complete diagnostic arthroscopy was first performed for every patient in this study to ensure that a PCL tear was present and to examine for other possible findings, such as meniscal or chondral injury in the knee. The double-bundle PCL reconstruction procedure was standardized; two femoral bone tunnels and one tibial tunnel were created at anatomical locations (Fig. 4)^16,17^. The femoral tunnel bridge between the anterolateral and posteromedial bone tunnel on the femur averaged 2.2 mm (range 1–3 mm). The ipsilateral semitendinosus tendon and gracilis tendon were harvested in all cases. The harvested grafts were trimmed at tripled- or quad-looped semitendinosus tendon for ALB and gracilis tendon for PMB, with a length of 70 mm used for PCL grafts. After careful preparation of grafts, the diameters of grafts were determined with sizer, followed we created the bone tunnels with size-matched drill. The EndoButton-CL (Smith & Nephew, Andover, MA) was attached on the femoral side. The baseball glove suture with a ULTRABRAID (Smith & Nephew, Andover, MA) was performed on the tibial side to secure the graft. The Endobutton was flipped on the femoral cortical surface. An assistant surgeon simultaneously applied tension using the tensiometer, and both the AL and PM grafts were fixed at 90° of knee flexion to the tibia^40^ using a double-spike plate system (Meira, Aichi, Japan) (Fig. 5).

### Rehabilitation

The rehabilitation protocol was unified in all cases as follows. The knee was immobilized at 20 degrees of flexion with a knee brace in each case. Range of motion exercises were performed gradually; 0°–60° of knee motion were started with a locked hard brace, a BREG X2K brace (Breg Inc, East Carlsbad, CA), for 1 week, and flexion of less than 90° was allowed until 6 weeks. At 6–8 weeks after surgery, flexion of less than 120° was allowed. Partial weight-bearing was started 1 week following surgery, with full weight-bearing at 4 weeks. Deep knee flexion, such as over 120°, and hamstring muscle exercise were prohibited until 4–5 months after surgery. The knee brace was used in all patients for the first 6 months after surgery. At 5–6 months after surgery, the gradual introduction of jogging and running was allowed, followed by a return to cutting actions and sports-specific drills at no sooner than 10 months postoperatively.

### Measurements

**Figure 6:**
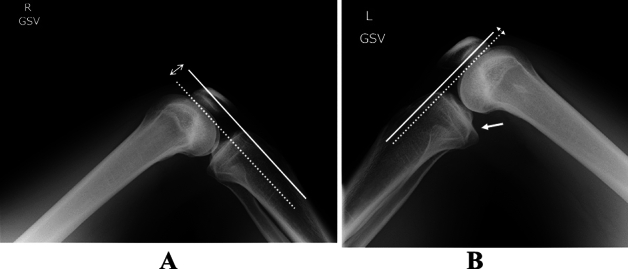
The gravity sag view X-ray examination. The side-to-side difference of the tibial-femoral step off, which was the interval between the tibial and femoral lines (two-way arrow), was defined as the posterior tibial translation. Dotted line as femoral line, and white line as tibial line. (**A**) Normal knee. (**B**) PCL-injured knee. Posterior tibial translation is observed (arrow).

The bilateral lateral radiographs with both hips at 45° and both knees kept upright at 90° of flexion were obtained for posterior laxity evaluation. GSV X-ray examinations according to Shino’s method^20^ were performed such as the measurement of side-to-side differences of the tibia-femur step off (Fig. 6).

According to Andrews report, PTT was stratified as grade 1 (< 5 mm), grade 2 (5 to < 10 mm), and grade 3 (over 10 mm)^45^. Compared with the results of bilateral GSV at 12 months after surgery, PTT of greater than 5 mm (grade 2 and 3) was defined as a ‘failure case’ in the present study, and categorized into ‘group F’, while less than 5 mm (grade 1) cases were defined as a ‘good’ group, and categorized into ‘group G’. These standardized lateral radiographs were also employed for measuring the posterior slope angle of tibia according to a previously validated method^46^.

Age, sex, body mass index (BMI), preoperative and 12 months postoperative GSV, posterior slope angle of the tibia, ALB and PMB graft diameters, and tibial tunnel diameter were evaluated. Complications such as meniscal injury and periarticular fracture were also evaluated. Values of peak isokinetic quadriceps and hamstring torque at 60°/s were measured with a Biodex-4 (Biodex Medical Systems Inc., Shirley, NY). Isokinetic peak torque values are presented as the leg symmetry index, with involved side/non-involved side as 100%. Three experienced senior orthopaedic surgeons performed these radiographic and clinical examinations and collected the data 12 months after surgery.

### Statistical analysis

Interobserver variability for the GSV value and posterior slope angle of tibia were satisfactory; mean intraclass correlation coefficient were 0.82 and 0.84, respectively. Statistical comparisons between the two groups by sex and the presence of complications were performed using the Fisher’s exact test and the 2 × 2 chi-squared test. Other potential risk factors were analysed with Spearman’s rank correlation coefficient and the Mann Whitney U-test. Multivariate logistic regression analyses were performed to identify significant risk factors according to Tachibana et al.^19^ and ORs and 95% CIs were calculated. ROC curve analysis was performed to identify the optimal cutoff value for postoperative PTT between subgroups. Statistical analyses were conducted using the statistical software package SPSS Statistics (Version 28; IBM Inc) and JMP Pro Version 16.0.0 (SAS institute). Statistical significance was indicated as p < 0.01 for correlation with postoperative PTT, and as p < 0.05 for all other comparisons. To evaluate the power, post hoc tests were conducted for factors that showed significant differences. As a result, powers for the femoral ALB tunnel diameter and posterior slope angle of the tibia were 0.735 and 0.905 (α = 0.05).

## Conclusion

Smaller ALB graft diameter and decreased posterior slope angle of tibia were considered risk factors for postoperative PTT. When the harvested hamstring autografts were smaller, harvesting an additional graft source to make a larger size ALB graft or converting the surgical procedure from double-bundle PCL reconstruction to other procedures should be considered.

## Data Availability

The datasets used and/or analyzed during the current study are available from the corresponding author on reasonable request.

## Abbreviations

PCL: Posterior cruciate ligament
ALB: Anterolateral bundle
PMB: Posteromedial bundle
ACL: Anterior cruciate ligament
PTT: Posterior tibial translation
GSV: Gravity sag view
OR: Odds ratio
CI: Confidence interval
ROC: Receiver operating characteristic
BPTB: Bone-patellar tendon-bone
BMI: Body mass index
